# Impact of Calcium and Phosphorus Levels on Optical Deterioration in Primary and Secondary Intraocular Lens Calcification

**DOI:** 10.1167/tvst.13.10.18

**Published:** 2024-10-10

**Authors:** Leoni Britz, Maximilian Hammer, Grzegorz Łabuz, Agnieszka Zielinska, Fabian Jester, Jan Freudenberg, Uwe Bunz, Christian Scholz, Gerd Uwe Auffarth, Timur Mert Yildirim

**Affiliations:** 1 The David J. Apple Laboratory for Vision Research, Heidelberg, Germany; 2 Department of Ophthalmology, University Hospital Heidelberg, Germany; 3 Institute of Physics, Faculty of Physics, Astronomy and Informatics, Nicolaus Copernicus University, Torun, Poland; 4 Organic Chemistry Institute, University of Heidelberg, Heidelberg, Germany; 5 Institute of Earth Sciences, University of Heidelberg, Heidelberg, Germany

**Keywords:** straylight, optical quality, intraocular lenses (IOLs), opacification, cataract surgery

## Abstract

**Purpose:**

The purpose of this study was to investigate the impact of calcium and phosphorus levels on optical deterioration in primary and secondary intraocular lens (IOL) calcification.

**Methods:**

A total of 18 explanted IOLs, 10 with primary, and 8 with secondary calcification, were examined. Straylight and light loss were evaluated as predictors of optical impairment. The individual amount of calcium and phosphorus was determined using thermogravimetry followed by emission spectroscopy (ICP-OES). The relationship between calcification and optical impairment was investigated.

**Results:**

Primary calcified IOLs contained significantly higher amounts of calcium and phosphorus compared to secondary calcified IOLs (calcium *P* < 0.02 and phosphorus *P* < 0.01), translating to greater light loss and significantly higher straylight mean values. In secondary calcification, light loss and straylight were highly dependent on calcium (*r*² = 0.90, *P* < 0.001 and *r*² = 0.70, *P* < 0.01) and phosphorus (*r*² = 0.66 and *r*² = 0.65, both *P* < 0.02), whereas these correlations were much lower in primary calcification (all *r* = 0.25, *P* > 0.05).

**Conclusions:**

ICP-OES is the first methodology to precisely assess the calcium and phosphorus content in IOL calcification thus based on mass ratios allowing improved molecular characterization. Primary calcification showed higher amounts of calcium and phosphorus, translating to higher straylight and light loss and thus a higher risk for impairment of visual quality than secondary calcification.

**Translational Relevance:**

This study is the first to quantify calcification and demonstrate the relationship to optical deterioration in IOLs, substantially contributing to understand how visual impairment arises in patients with calcified IOLs.

## Introduction

Calcification of hydrophilic acrylic intraocular lenses (IOLs) is a rare but well-known complication of cataract surgery.^1^^–^^5^ Its incidence varies significantly depending on several risk factors. Particularly elevated rates are observed following endothelial keratoplasty, where the incidence can reach 4.5%^6^ and in certain IOL models, where incidences can be as high as 14.5%^7^. IOL calcification is attributed to the formation of calcium phosphate crystals on and within the lens polymer.^8^^,^^9^ This crystal formation is irreversible, making lens exchange the only available treatment option. Calcification is specific to hydrophilic polymers, with hydrophobic acrylic polymers showing no signs of calcification.^8^^,^^10^^,^^11^ Hydrophilic acrylic materials have found various applications in ophthalmology. Used for phakic,^12^^,^^13^ pseudophakic posterior chamber,^14^^,^^15^ scleral fixated IOLs^16^^,^^17^, or artificial corneal endothelial transplants,^18^^,^^19^ they offer several advantages like flexibility in microincision surgery, higher uveal biocompatibility than hydrophobic polymers, and ease of alignment in toric IOLs.^20^^,^^21^ To date, calcification has only been reported in hydrophilic IOLs under specific conditions: primary calcification is linked to certain manufacturing processes,^22^^–^^25^ whereas secondary calcification is associated with risk factors that create a conducive environment for calcification to occur.^5^^,^^26^^–^^28^

So far, the process of calcification, precise risk factors, and the reasons why certain hydrophilic polymers are more prone to calcification than others remain incompletely understood.^1^^,^^5^^,^^26^ It has been proposed that phosphorus, present as phosphate in the aqueous humor, and calcium may precipitate to form unstable phases that hydrolyze into hydroxyapatite over time.^8^^,^^11^ However, this hypothesis has not been definitively proven yet. A comprehensive understanding of this process is crucial for preserving the advantages of hydrophilic acrylic polymers while minimizing the risk of calcification.^29^^,^^30^ Therefore, laboratory studies investigating various risk factors, IOL materials, and their susceptibility to calcification are imperative.^10^^,^^11^^,^^31^ To achieve this, quantification of calcification would be essential. However, until now, there is no method to objectively determine the amount of calcium and phosphorus in calcified IOLs. In this study, we established the first method to precisely quantify the calcifications in a large number of naturally calcified donor IOLs and explored the relationship between calcium and phosphorus levels and visual impairment.

## Methods

### Light Microscopy Examination

A total of 18 IOLs, 10 cases of primarily calcified IOLs (Lentis L-313; Teleon Surgical GmbH, Germany), and 8 cases of secondarily calcified IOLs (CT Asphina 409M; Carl Zeiss Meditec AG, Germany) were examined. These IOLs were sent to the explanted biomaterials register of the David J. Apple Laboratory for Ocular Pathology Laboratory after explantation due to calcification. An unused IOL of each model was used as a negative control (*n* = 20). Initially, light microscopy was performed according to a standardized, established protocol^9^^,^^27^^,^^32^^,^^33^ to examine the distribution of calcification, which was used to ensure differentiation between homogeneous (primary) and localized (secondary) calcification. The medical information provided by the surgeons explanting the IOLs were reviewed to determine the duration of implantation and any intraocular surgeries performed after IOL implantation.

### Straylight Measurement

A straylight meter (C-Quant; Oculus Optikgeräte GmbH, Germany) and an established set up^34^^–^^37^ was used to quantify light scattering caused by intraocular lens calcification. The lens under investigation was securely positioned in an IOL holder with a 5.5 mm aperture simulating the pupil and was subsequently placed in front of the device. An adapter, containing a glass cuvette filled with balanced saline solution (BSS), was used to align the IOL along the optical axis of the C-Quant. A plano-convex lens projected the C-Quant test field onto the IOL. The straylight source was obstructed by a diaphragm positioned in front of the cuvette, allowing only the scattered light produced by the IOL to be perceived by the examiner. Straylight measurements were conducted twice for each lens, and mean values were calculated. The results were adjusted for the straylight generated by the setup and the negative control lens. All measurements were conducted by the same examiner (author L.B.), who was blinded to the sample. The C-Quant is a thoroughly validated method for measuring straylight, demonstrating high repeatability and reproducibility in both in vivo and in vitro settings.^34^^,^^38^^,^^39^

### Light Loss Evaluation

A power meter (PM100D; Thorlabs Inc., Newton, NJ, USA) and an optical bench setup (Optispheric IOL Pro 2 optical bench; Trioptics GmbH, Jena, Germany) were used to measure light transmittance of the opacified lenses. The specimen was placed into a plastic custom insert in a watertight container with a glass bottom. BSS was instilled and the container was closed with a glass lid, while taking care to avoid bubbles inside. A preset of optical bench setup was used, measuring light transmittance of the central 3 mm of the lens optic using an aperture and at a wavelength of 546 nm, which closely aligns with the peak sensitivity of the human retina. The setup light transmittance was measured and the preset calibrated. Each lens was measured twice by a single blinded operator (author A.Z.) and the results were averaged. Light loss of the lenses was calculated in the percentage (%) of the new lens light transmittance.

### Thermogravimetric Analysis 

For quantification of calcium und phosphorus content, eight lenses each of primary and secondary calcification were evaluated. Before measurement, the IOLs were carefully rinsed with bidistilled water. Then, thermogravimetric analysis (TGA) was used to obtain the inorganic components of the specimens by burning under standardized conditions. The analyses were performed using a TGA/DSC 1 device (Mettler Toledo GmbH, Giessen, Germany) under ambient conditions in an aluminum oxide crucible (70 µL). The measurements were carried out in a range of 25 to 900°C with a heating rate of 10 kmin^−1^.

### Inductively Coupled Plasma Optical Emission Spectroscopy 

**Figure 1.**
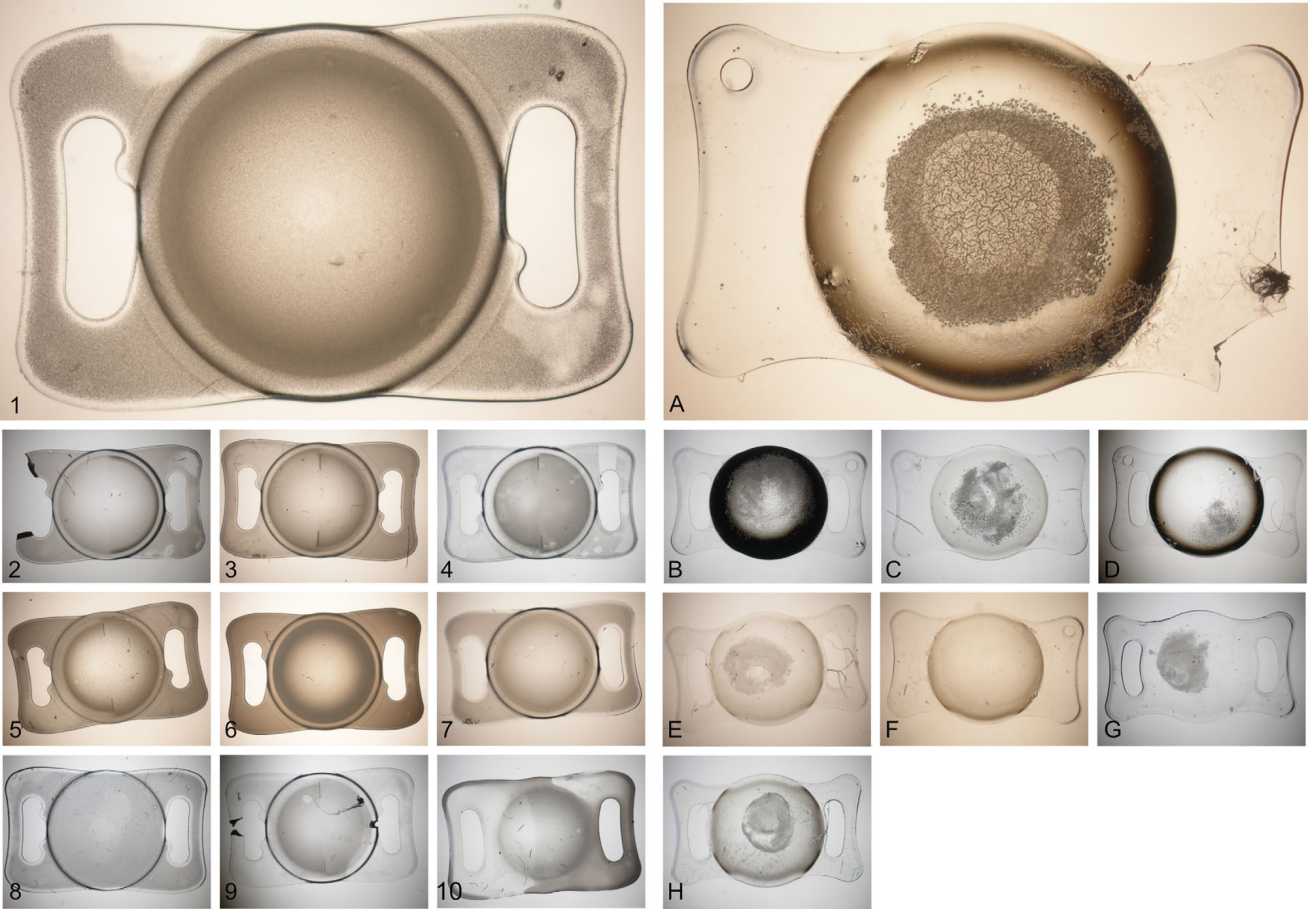
Primary calcification (1–10) shows homogeneous crystal formation over the entire lens optic and haptics. Secondary calcification (**A–H**): In these cases, a secondary surgery with intraocular gas injection led to crystal formation limited to circular areas of the central optic. Some IOLs were damaged during the removal surgery, however, all tested IOLs had an intact optic center.

To determine calcium and phosphorus amount in the ashes of the specimens, inductively coupled plasma optical emission spectroscopy (ICP-OES) was used. Before the measurements could be carried out, the ash samples must be digested. For this, the TGA crucibles containing the ash of the samples, along with their lids, were transferred into a digestion tube (Digitube 50 mL; SPC Science, France). In this digestion tube, 2 mL of 65% p.A. double sub-boiled Suprapur nitric acid and 250 µL of 30% p.A. hydrogen peroxide (H_2_O_2_) were added to dissolve the ash. The mixture was heated up to 90°C in a graphite block for 1 hour. Subsequently, the solutions were transferred to 15 mL centrifuge tubes (Sarstedt, PP), the crucibles were rinsed, and the solution was filled up to 10 mL with Milli-Q water. Scanning electron microscopy with energy-dispersive X-ray spectroscopy investigations of the crucibles were used to ensure a complete dissolution of the ashes was achieved. Following this, the measurements were conducted using ICP-OES (ICP-OES 720; Agilent Technologies, USA). To analyze the digestion solutions, a wavelength of 317.933 nm was used for calcium and 213.618 nm for phosphorus. For each specimen, triplicate measurement values were obtained, and the means were used. Results were corrected by the value of the new control lenses. We had to exclude one secondary calcified IOL (Fig. 1H) from the quantitative measurements due to sample contamination in the process.

### Statistical Analysis

Statistical analysis was carried out using Prism version 10.2.2 (GraphPad Software, Boston, MA, USA). Normality was evaluated using the Kolmogorov-Smirnov test. Pearson correlation, student's *t*-test, and bivariate linear regression were applied.

## Results

Figure 1 shows microscopic evaluation of the calcification pattern and crystal distribution in the lenses. The mean duration of implantation was M = 8.55 years (standard deviation [SD] = 1.99) for cases with primary calcification and M = 7.92 years (SD = 2.52) for cases with secondary calcification. In all cases of secondary calcification, surgeries involving intraocular gas injection were performed after IOL implantation (4 IOLs with Descemet membrane endothelial keratoplasty, and 4 IOLs with Pars-plana-vitrectomy with gas injection). Assuming gas exposure as a major trigger for calcification, secondary calcified lenses were explanted at M = 3.67 (SD = 4.03) years after the surgery involving gas installation.

### Straylight Measurement

**Figure 2.**
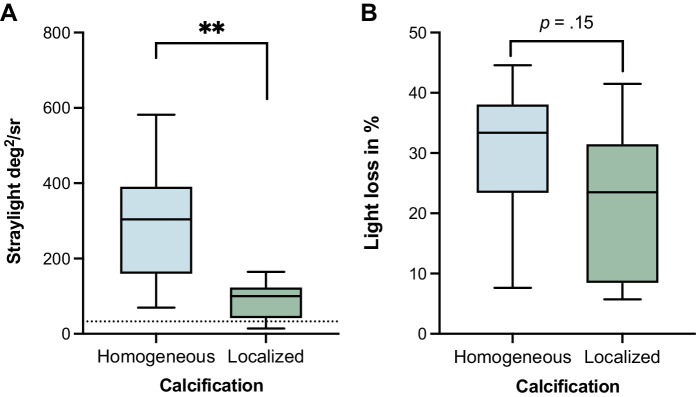
Straylight (**A**) and light loss caused by calcification in percentage (%) of the negative control lens (**B**) in primary and secondary calcification. Median with interquartile range, *P* value. Primary calcification shows higher straylight and higher light loss than secondary calcification. The dotted line in **A** represents mean straylight level of a cataractous lens for reference.

Primary calcification showed significantly higher mean straylight values (M = 289.28 deg^2^/sr, SD = 162.24) than secondary calcification (M = 88.88 deg^2^/sr, SD = 50.64, *P* < 0.001; Fig. 2A). In both groups, the straylight was strongly increased compared to the mean straylight of the control lenses (Oculentis LS 313 for primary calcification = 2.97 deg^2^/sr and CT Asphina 409M for secondary = 3.87 deg^2^/sr) and a cataractous lens (s = 33.1 deg^2^/sr).^40^

### Light Transmittance

The mean light loss in lenses with primary calcification was higher than in lenses with secondary calcification but contrary to straylight levels, this difference was not statistically significant (Fig. 2B, *P* = 0.15). In primary calcified lenses, an average of 30.48% of light was lost due to calcification, whereas secondary calcification caused a light loss of 22.09% of the control lens. High positive correlations for straylight and light loss were found for both primary (*r* = 0.70, *P* < 0.05) and secondary calcification (*r* = 0.93, *P* < 0.01).

### Quantification of Calcium and Phosphorus Using TGA and ICP-OES

**Figure 3.**
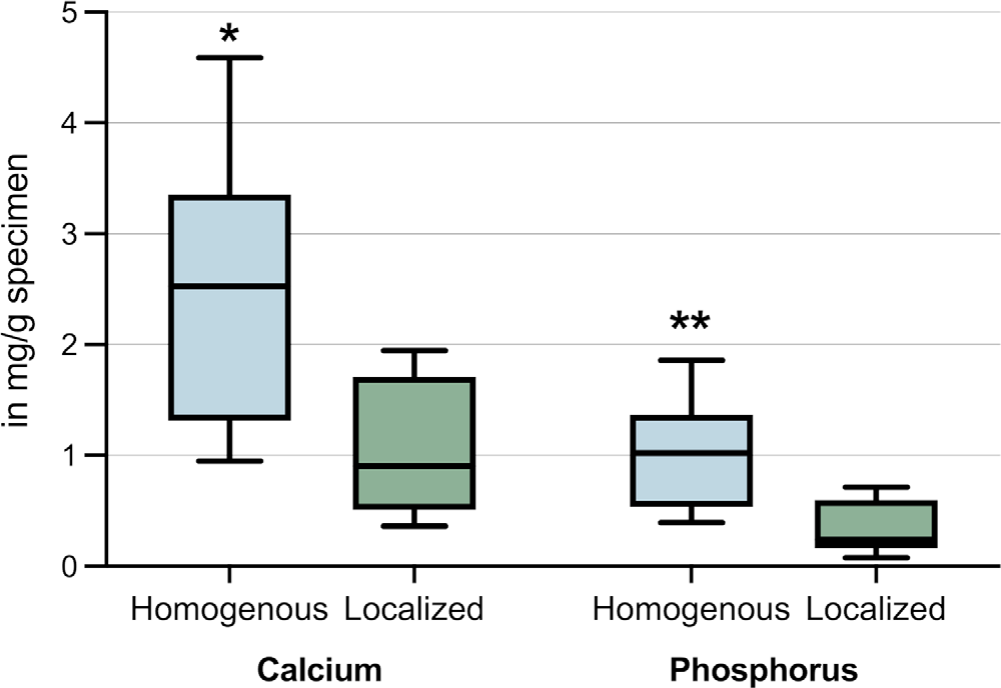
Calcium und Phosphorus content of the specimens as obtained by ICP-OES. Median with interquartile range, *P* value. Primary, homogenous calcification showed significant higher calcium and phosphorus amounts than secondary, localized calcification (calcium *P* < 0.05 and phosphorus *P* < 0.01).

**Figure 4.**
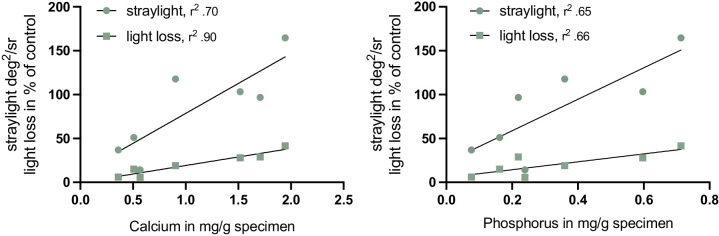
Linear regression model. Induced straylight and light loss in secondary, localized calcification are significantly dependent on the amount of calcium (*P* < 0.01 and *P* < 0.001, respectively) and phosphorus (both *P* < 0.02) within the central lens optic.

In line with the straylight and light loss results, significant higher amounts of calcium and phosphorus were found on average in primary calcification, compared to secondary calcification (calcium *P* < 0.02, phosphorus *P* < 0.01; Fig. 3). In secondary calcification, light loss and straylight significantly depended on calcium (*r^2^* = 0.90, *P* < 0.001 and *r^2^* = 0.70, *P* < 0.01) and phosphorus (*r^2^* = 0.66 and *r^2^* = 0.65, both *P* < 0.02) amount in the specimens (Fig. 4), whereas in primary calcification the correlations were rather low (all *r* = 0.25, *P* > 0.05). Looking at the mass ratio of calcium to phosphorus (Ca/P) in the explanted lenses, it is noticeable that both primary (median Ca/P = 2.45, [2.42, 2.47]) and secondary calcified lenses (median Ca/P = 2.73, [2.68, 3.95]) showed higher mass ratios than that of hydroxyapatite (Ca/P = 2.15).

Interestingly, the calcium and phosphorus content in cases of secondary calcification were strongly and statistically significantly related to the time after surgery with intraocular gas instillation (calcium *r*^2^ = 0.89, *P* < 0.02 and phosphorus *r*^2^ = 0.83, *P* < 0.05). In contrast, for primary calcification, moderate but statistically nonsignificant relationships were observed (calcium *r*^2^ = 0.58, *P* = 0.24 and phosphorus *r*^2^ = 0.56, *P* = 0.25).

## Discussion

In summary, we established the first ever method for the exact quantification of the calcification in intraocular lenses differentiating between phosphorus and calcium. Further, we investigated the relationship to optical deterioration in primary and secondary calcification in naturally calcified IOL specimen explanted from patients.

Using a combination of TGA and ICP-OES, we determined both the calcium and phosphorus content in calcified hydrophilic acrylic IOLs. In one of our previous studies, Łabuz et al. evaluated the size and number of calcium granules in von Kossa-stained IOL sections and found a proportional relationship to calculated light loss and straylight in secondary calcification (*n* = 4).^40^ Expanding upon this, our study analyzed a larger sample size and the entire optic, demonstrating that light loss and straylight are significantly dependent on calcium and phosphorus content in secondary calcified lenses, reinforcing our initial findings.

Tripodi et al. used Instrumental Neutron Activation Analysis (INAA) to measure the calcium content of a single, secondarily calcified Hydroview H60M IOL, reporting 144 µg.^41^ In our study, calcium content ranged from 25.71 µg to 142.07 µg (mean = 72.67 µg) for primarily calcified IOLs, and from 9.62 µg to 57.05 µg (mean = 29.63 µg) for secondarily calcified IOLs. Our values were generally lower but within comparable orders of magnitude. Given that this is the first study to measure calcium and phosphorus in a larger sample of calcified IOLs, the observed discrepancies are likely due to variations in the extent of calcification rather than differences in measurement methodologies.

ICP-OES enhances the precision of phosphorus content determination in specimens.^42^ Evaluating calcium granule size and distribution only provides estimations regarding causal relationships between calcium-phosphorus levels and visual impairment.^40^ However, the destructive nature of the ICP-OES analysis presents a significant limitation. In contrast, INAA offers non-destructive, highly accurate sample analysis but yields unreliable outcomes for certain elements, such as phosphorus.^43^ Furthermore, the limited availability of neutron reactors and high operational costs present challenges for its implementation.^44^ Accurately determining the degree of calcification enables comparisons among different forms of calcification, lens materials, and associated risk factors. In our study, we investigated the calcium and phosphorus content in primary and secondary calcification and its association with optical impairment. Higher levels of calcium and phosphorus were observed in primary calcification, correlating with increased straylight and light loss values compared to secondary calcification. Consistent with prior studies,^45^ these findings suggest a greater potential for glare-related visual impairment in primary calcification compared to secondary calcification.

Another interesting finding of our investigation was that the Ca/P mass ratio in our samples exceeded that of hydroxyapatite. To date, only hydroxyapatite has been identified in calcified IOLs.^9^^,^^11^ However, it has been hypothesized that hydroxyapatite formation occurs through precursor phases.^8^ Phosphorus, in the aqueous humor present as phosphate, and calcium may precipitate to form amorphous calcium phosphate, an unstable intermediate phase, which hydrolyzes over time to hydroxyapatite.^8^^,^^31^ The results obtained in our study support this hypothesis. Amorphous calcium phosphate demonstrates a variable Ca/P mass ratio ranging from 1.55 to 2.84, surpassing the stoichiometrically pure hydroxyapatite with a Ca/P ratio of 2.15.^46^ In our samples, a median Ca/P ratio of 2.47 (primary calcification) and 2.73 (secondary calcification) was observed. These findings suggest that some of the calcium phosphate crystals present in the lenses may exist as amorphous calcium phosphate, which may subsequently undergo maturation into octacalcium phosphate or hydroxyapatite over time.^47^ The impact of this crystal formation on other lens properties, such as mechanical and structural characteristics, has not yet been investigated. Future studies should explore the consequences of pronounced calcification.

Using ICP-OES, we examined calcium and phosphorus levels in primary and secondary calcified lenses. We found higher amounts of calcium and phosphorus in primary calcification compared to secondary calcification (see Fig. 3). This difference may be attributed to primary calcification affecting a larger volume of the lens polymer than secondary calcification.^27^^,^^32^^,^^48^^–^^51^ In primary calcified lenses, crystal formation appeared uniform across the lens optics and haptics, whereas secondary calcification was limited to a central area of the IOL optic (see Fig. 1). It is important to note that both lens types investigated in this study are made from the same hydrophilic acrylic starting material, suggesting that this contrast is most likely due to variations in exposure to risk factors. Rinsing with phosphate-containing solutions during the manufacturing process in primary calcification leads to an increased phosphorus content throughout the entire lens polymer. This most likely results in the precipitation of calcium phosphates across the entire IOL.^1^^,^^23^ Contrarily, in secondary calcification associated with intraocular gas instillation, it is speculated that the capsular bag shields the peripheral optic and haptic of the IOL from gas-induced dehydration,^26^ restricting crystal formation to a circular portion corresponding to capsulorhexis.^52^^,^^53^ Another factor potentially contributing to elevated calcium and phosphorus levels is the implantation duration.^24^^,^^26^^,^^54^^,^^55^ For secondary calcification, calcium and phosphorus content strongly and significantly increased in relation to implantation time following surgery with gas instillation. Extended duration of implantation facilitates the diffusion of calcium and phosphorus into the lens polymer, leading to increased precipitation and calcium phosphate formation.^30^ In contrast, for primary calcification, the relationship was not statistically significant. There was greater variability in the data within this group, and additional studies are needed to further analyze the relationship.

Patients with calcified IOLs commonly experience significant glare, reduced contrast vision and poor vision in low light conditions,^27^^,^^34^^,^^35^^,^^56^ with visual acuity being reduced only in severe cases.^45^ In our study, we evaluated straylight and light loss in primary (homogeneous) and secondary (localized) calcification to predict optical impairment.^45^^,^^57^ Using an in vitro setup, we correlated these optical parameters with calcium-phosphorus content for the first time. Increasing amounts of calcium and phosphorus were associated with higher light loss and significantly elevated straylight levels in primary homogeneous calcification compared to secondary localized calcification.^45^^,^^58^ These findings suggest a causal relationship between straylight and calcium-phosphorus content.

**Figure 5.**
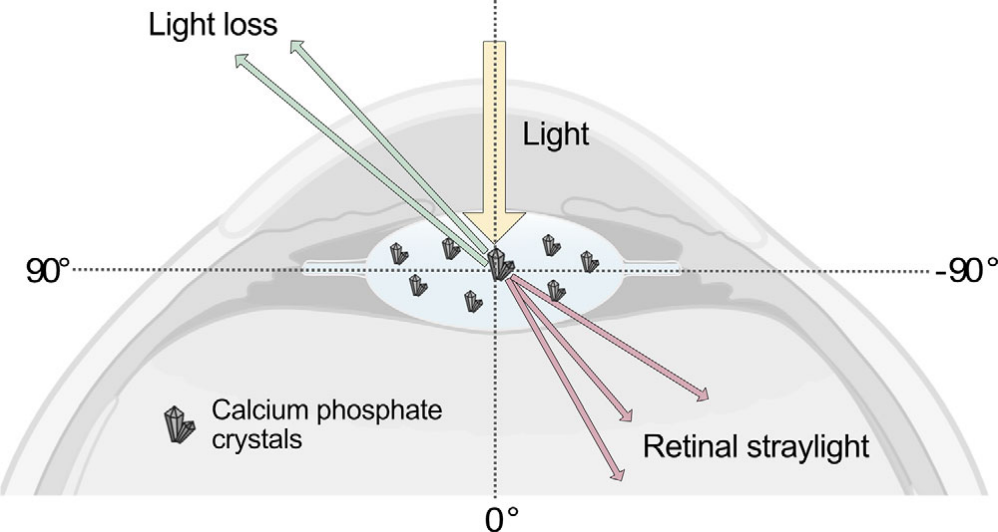
Schematic representation of light scattering by calcium phosphate crystals in the lens polymer. Light scattered at angles ±90 degrees leads to retinal straylight, while light scattered at angles > ±90 degrees contributes to light loss. Straylight can result in symptoms such as glare, whereas light loss can reduce contrast vision and vision in low-light conditions.

In cases of lens calcification, some incident light passes through the lens optics unhindered, whereas another fraction is scattered by calcium and phosphorus granules.^40^ When scattering occurs at an angle ± 90 degrees, the scattered light falls toward the retina, leading to retinal straylight (Fig. 5). Conversely, scattering angles > ±90 degrees prevent light from entering the eye, contributing to light loss.^59^ The greater the calcium and phosphorus content in the central optic, the more pronounced the light scattering at the granules, leading to elevated and correlated straylight and light loss values. Our study focused on assessing straylight and light loss within the central lens optic, respectively. This may explain the lack of significant correlations observed in primary calcification, where calcium phosphorus granules are also present in the peripheral optic and lens haptics. In contrast, secondary calcification primarily affects the region of the lens optic assessed for visual quality (see Fig. 1).

However, given the higher absolute values in scattered light and light loss, it can be inferred that primary calcification poses a greater risk for glare-related visual impairment. The depth of crystal formation within the lens polymer in primary compared to secondary calcification was not investigated in this study. Future research is needed to determine whether different depths of crystal formation influence light scattering and light loss.

Fortunately, reports of primary calcification in entire IOL batches have become less frequent due to changes in manufacturing processes.^23^^,^^24^ In contrast, cases of secondary calcification related to additional intraocular surgery have increased.^6^^,^^54^ In a subgroup of patients, calcification could be prevented by implanting hydrophobic acrylic lenses. However, for cases where hydrophilic acrylic materials are preferred, like toric or sutureless scleral lens implantations or for patients who benefit from higher biocompatibility,^20^^,^^21^^,^^60^ IOL calcification remains a challenge. Natsi et al. introduced a novel approach involving the coating of hydrophilic IOLs with reduced graphene oxide as a potential method for preventing intraocular lens calcification.^29^ However, these reduced graphene oxide coated lenses have not been sufficiently investigated to be used in clinical practice, and further research is needed to determine if they represent a viable solution for preventing lens calcification.

In conclusion, we have established the first method for measuring calcium and phosphorus content in IOL calcification, enabling comparison of calcification in different lens materials and risk factors. Our findings demonstrate that the impairment of optical function in calcified lenses, assessed through straylight and light loss, primarily correlates with the amount and distribution of calcium and phosphorus within the lens optic. Specifically, our study indicates that primary, homogeneously calcified lenses exhibit a greater potential for visual function impairment compared to secondary and localized calcification. These findings substantially contribute to understand how visual impairment arises in patients with calcified IOLs and show that straylight and light loss can be used as measures to estimate the degree of calcification within the lens optic.
